# *De novo* TANGLED1 recruitment from the phragmoplast to aberrant cell plate fusion sites in maize

**DOI:** 10.1242/jcs.262097

**Published:** 2024-06-19

**Authors:** Aimee N. Uyehara, Beatrice N. Diep, Lindy A. Allsman, Sarah G. Gayer, Stephanie E. Martinez, Janice J. Kim, Shreya Agarwal, Carolyn G. Rasmussen

**Affiliations:** Department of Botany and Plant Sciences, Center for Plant Cell Biology, University of California Riverside, CA 92521, USA

**Keywords:** Preprophase band, Division, Phragmoplast, Mitosis, Cytoskeleton, Maize

## Abstract

Division plane positioning is crucial for proper growth and development in many organisms. In plants, the division plane is established before mitosis, by accumulation of a cytoskeletal structure called the preprophase band (PPB). The PPB is thought to be essential for recruitment of division site-localized proteins, which remain at the division site after the PPB disassembles. Here, we show that the division site-localized protein TANGLED1 (TAN1) is recruited independently of the PPB to the cell cortex by the plant cytokinetic machinery, the phragmoplast, from experiments using both the PPB-defective mutant *discordia1* (*dcd1*) and chemical treatments that disrupt the phragmoplast in maize. TAN1 recruitment to *de novo* sites on the cortex is partially dependent on intact actin filaments and the myosin XI motor protein OPAQUE1 (O1). These data imply a yet unknown role for TAN1 and possibly other division site-localized proteins during the last stages of cell division when the phragmoplast touches the cell cortex to complete cytokinesis.

## Teaser

**Highlighted Article:** The plant division site protein TANGLED1 is recruited to *de novo* cell plate insertion sites independently of the preprophase band.

## INTRODUCTION

In typical land plant cell divisions, two cytoskeletal structures participate in division plane positioning: the PPB, which assembles during late G2, and the phragmoplast, which assembles during telophase and expands to complete cytokinesis. The PPB is a transient cortical ring of microtubules and actin that is an early indicator of the cell division plane (Pickett-Heaps and Northcote, 1966; Kakimoto and Shibaoka, 1987; Mineyuki, 1999; Smertenko et al., 2017). Following chromosome and organelle redistribution in metaphase and anaphase, the phragmoplast forms to facilitate cell plate formation, which divides the two daughter cells (Gunning, 1982; Samuels et al., 1995; Müller and Jürgens, 2016). The location where cytokinesis is completed is the cell-plate fusion site, and if the cell plate fuses at the location previously marked by the PPB, that location is called the division site (Smertenko et al., 2017).

Genetic disruption of PPB formation often leads to significantly stunted growth, division plane positioning defects and disrupted cortical microtubule organization, which might impede cell expansion (Whittington et al., 2001*;* Torres-Ruiz and Jürgens, 1994; Camilleri et al., 2002; Kawamura et al., 2006; Azimzadeh et al., 2008; Wright et al., 2009; Drevensek et al., 2012; Kirik et al., 2012; Spinner et al., 2013; Kumari et al., 2021; Muroyama et al., 2023). However, even the absence of >80% of PPBs generates macroscopically normal plants with minor division plane orientation defects that have been attributed to spindle-positioning defects (Ambrose and Cyr, 2008; Schaefer et al., 2017).

PPB formation requires the PROTEIN PHOSPHATASE TYPE 2A (PP2A) B″ regulatory subunit encoded by two related genes in maize called *discordia1* (*dcd1*) and *alternative discordia1* (*add1*) (Gallagher and Smith, 1999; Wright et al., 2009), homologs to *fass* (also known as *ton2*) in *Arabidopsis* (Torres-Ruiz and Jürgens, 1994; Camilleri et al., 2002). In *A. thaliana* FASS forms a complex with microtubule-binding proteins including TONNEAU1, TONNEAU1-RECRUITING-MOTIF proteins and other PP2A subunits that disrupt cortical microtubule organization and PPB formation (Wright et al., 2009; Spinner et al., 2013). Although *dcd1 add1* double mutants are seedling lethal and never form PPBs, single *dcd1* mutants grow well and do not have PPB formation defects in symmetrically dividing cells (Wright et al., 2009). Instead, *dcd1* single mutants produce defective PPBs in several asymmetrically dividing cells such as the grass-specific stomatal complex subsidiary cells, leading to division positioning defects. Subsidiary cells, generated from an asymmetric division, serve as an excellent model to analyze division-plane orientation due to consistently positioned divisions and well-characterized signaling pathways (Spiegelhalder and Raissig, 2021; Gray et al., 2020).

The PPB serves as a hub to recruit multiple proteins, including a small subset of proteins that remain at the division site after PPB disassembly. One division-site localized protein, TANGLED1 (TAN1), binds microtubules and is required for properly oriented divisions (Smith et al., 1996, 2001; Martinez et al., 2017, 2020). TAN1 localization to the division site requires an intact PPB, where it is maintained until cytokinesis is completed (Walker et al., 2007; Rasmussen et al., 2011; Martinez et al., 2017). In maize, TAN1 also colocalizes with the phragmoplast midline (Martinez et al., 2017). The maize *tan1* mutant has mostly normally placed PPBs, but phragmoplast guidance defects lead to misoriented symmetric and asymmetric divisions (Smith et al., 1996; Martinez et al., 2017, 2020). TAN1 promotes contact angle-independent microtubule interactions, which guide the phragmoplast to the division site (Bellinger et al., 2023; Martinez et al., 2020).

Here, we use the partially defective PPBs in *dcd1* mutants to measure the contribution of PPB formation to division plane positioning. To our surprise, and contrary to previous reports, the *dcd1* mutant revealed an unexpected *de novo* recruitment of TANGLED1 from the phragmoplast to misoriented cell plate fusion sites. We demonstrate that *de novo* TAN1 accumulation occurs in multiple mutants and chemically treated cells that have division plane positioning defects. Furthermore, TAN1 accumulation is partially dependent on actin and the myosin XI protein OPAQUE1 (O1).

## RESULTS AND DISCUSSION

### Defects in *dcd1* mutant PPB formation reduce TAN1–YFP accumulation

**Fig. 1.**
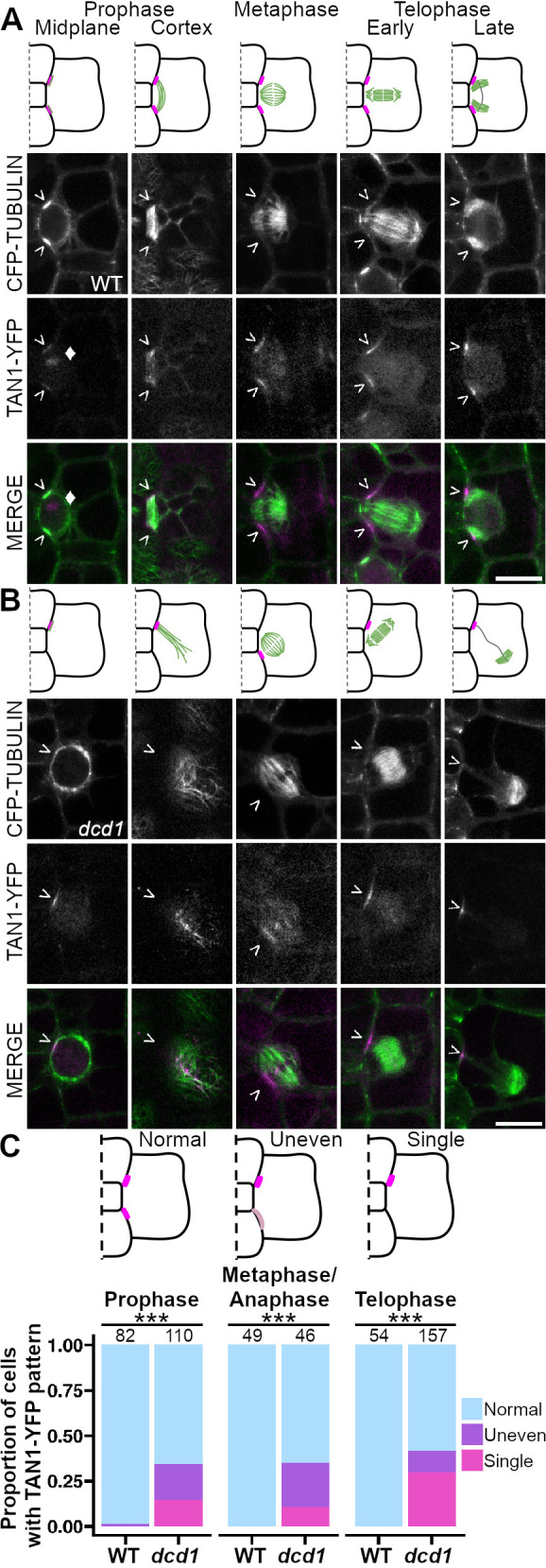
**PPB formation and TAN1–YFP recruitment is defective in *dcd1* mutants.** (A,B) Model of (A) wild-type (WT) or (B) *dcd1* mutant subsidiary cell divisions. Cell walls (black), microtubule structures (green), and TAN1–YFP (magenta) are shown. Below are representative images with CFP–TUBULIN labeling microtubules (green) and TAN1–YFP (magenta) labeling the division site (>) and sometimes the nucleolus indicated with a diamond (♦). (C) Observed TAN1–YFP accumulation patterns. Darker and lighter shades of magenta represent higher and lower TAN1–YFP intensities reflecting greater or less accumulation, respectively. Below, stacked bar plot comparing wild-type and *dcd1* mutant cells that exhibit various TAN1–YFP patterns represented by the schematic models above. Numbers above bars represent cells examined. ***P<0.001 (Fisher's exact test). *N*=19 wild-type plants and 7 *dcd1* mutant plants. Scale bars: 10 μm.

To determine whether partially defective PPBs affect TAN1 recruitment to the division site, we observed TAN1–YFP in *dcd1* mutants and wild-type siblings with the microtubule marker CFP–TUBULIN (Martinez et al., 2017). Wild-type subsidiary cells had no defects in PPB formation or TAN1–YFP accumulation (*n*=0/112 cells from 19 plants, Fig. 1A; Fig. S1A). In contrast, *dcd1* mutant cells often had defective PPBs that incompletely encircled the cell, similar to previous results (∼40%, Wright et al., 2009) (38%, *n*=42/110 cells from 7 plants). Defective PPBs had uneven microtubule accumulation, including one-sided accumulation (‘singular’, Fig. 1B; Fig. S1B). Correspondingly, uneven or singular TAN1–YFP accumulation at the division site was observed in preprophase and prophase (35% *n*=38/110 from 7 *dcd1* plants), in metaphase and anaphase (35%, *n*=16/46), and in telophase (41%, *n* =65/157, Fig. 1C), suggesting that PPB establishment is required for TAN1 recruitment to the division site.

### Defective PPBs in *dcd1* mutants cause division plane positioning defects

**Fig. 2.**
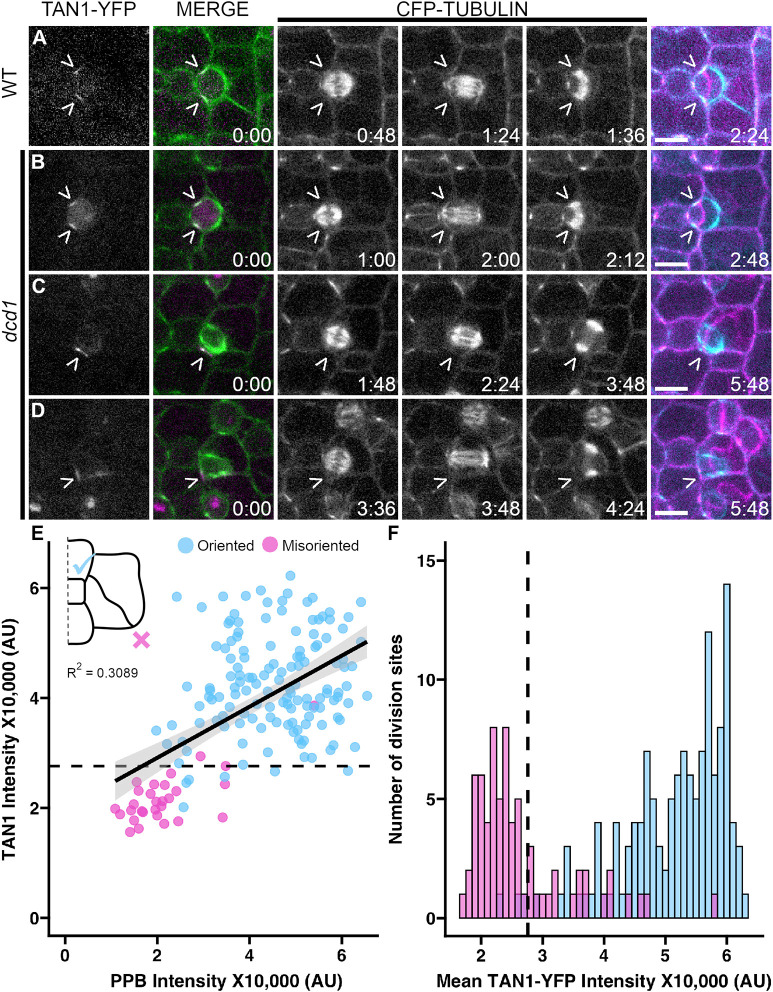
**Defective preprophase bands and TAN1 localization result in misoriented divisions.** Time-lapses of subsidiary cell divisions expressing *CFP-TUBULIN* and *TAN1-YFP* in (A) wild-type (WT) cells and (B–D) *dcd1* mutant cells. Left-most columns show TAN1–YFP localization at *t*=0 [merge shows both TAN1–YFP (magenta) and microtubules (green)]. The last column overlays the PPB in the first frame (cyan) and final division frame (magenta). Carets (>) mark the division site. Time stamps are in hours:minutes. Scale bars: 10 µm. (E) Comparative TAN1–YFP and PPB intensity from time-lapses of *dcd1* mutant cells. ‘Oriented’ describes phragmoplasts that return to the division site and ‘misoriented’ describes cell plate insertion at atypical locations. *n*=85 cells, *N*=4 plants. (F) Histogram displaying the mean TAN1–YFP fluorescence intensity of cell division sites in *dcd1* mutant cells colored by division orientation at the first timepoint for time-lapses that start after prophase. *n*=112 cells. *N*=4 plants. Dotted line represents the visible detection limit or the point at which TAN1–YFP fluorescence is distinguishable over background. For E and F, blue, oriented, magenta, misoriented. AU, arbitrary units.

Like many studies that have examined the role of the PPB in division plane positioning (Camilleri et al., 2002; Azimzadeh et al., 2008; Drevensek et al., 2012; Schaefer et al., 2017; Wang et al., 2019; Kumari et al., 2021), our initial analysis of *dcd1* mutants was performed using static images. This data generated strong correlative support for the role of the PPB in division plane positioning, but cell division trajectories were not analyzed. To directly assess the relationship between PPB formation, TAN1 accumulation and final division positioning, 12-min time intervals were used to track divisions, capitalizing both on the invariant positions of subsidiary cell divisions and the *dcd1* partial PPB formation defects (Fig. 2A–D). At *dcd1* subsidiary cell division sites (*n*=374 division sites total from 4 plants), we measured the TAN1–YFP and/or CFP–TUBULIN fluorescence intensities and classed final divisions as ‘oriented’ or ‘misoriented’ dependent upon whether the phragmoplast returned to the division site. Robust PPB microtubule accumulation strongly predicted correctly oriented cell divisions. Division sites with undetectable TAN1–YFP tended to be misoriented (79%, *n*=26/33 cells with TAN1–YFP fluorescence intensity at background levels, Fig. 2E). For cell divisions captured in later stages, 94% (metaphase, anaphase or telophase, *n*=50/53) of misoriented final divisions were associated with undetectable TAN1–YFP intensity at the time-lapse onset (Fig. 2F, *n*=112 cells). These data show that the PPB is essential for division plane positioning in subsidiary mother cell divisions and that TAN1–YFP localization at the division site is a reasonable proxy for previous PPB formation.

### TAN1–YFP accumulates at misoriented cell plate insertion sites

**Fig. 3.**
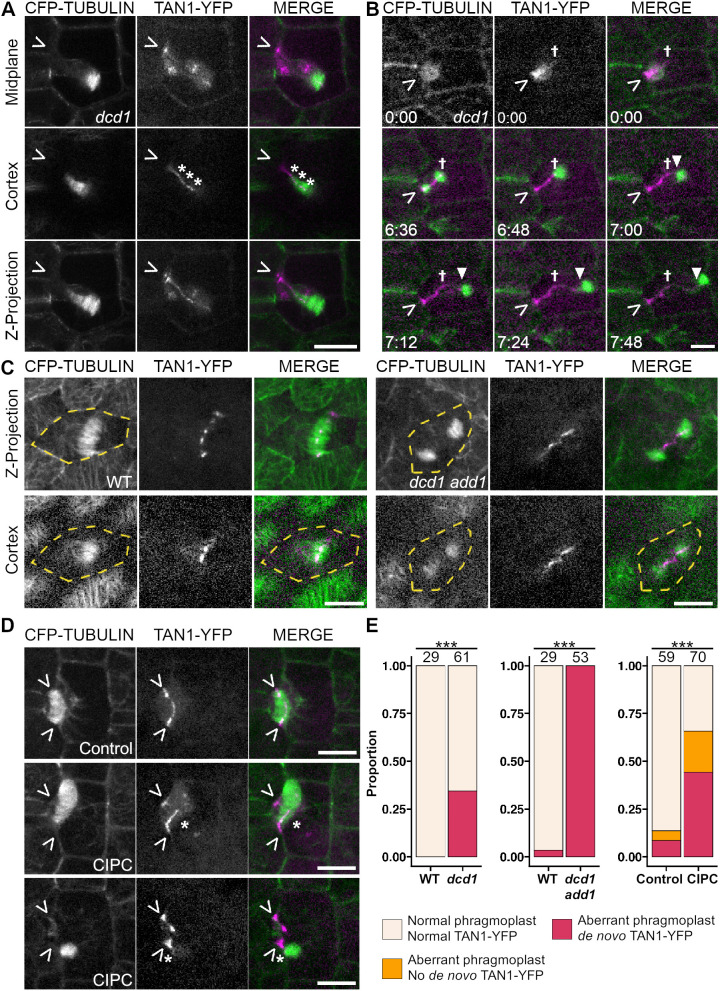
**Cell plate insertion sites accumulate *de novo* TAN1–YFP.** (A–D) CFP–TUBULIN (green) and TAN1–YFP (magenta) in various dividing cells. Carets (>) mark the division site and asterisks (*) mark *de novo* TAN1–YFP. (A) *dcd1* mutant subsidiary mother cell with *de novo* cortex-localized TAN1–YFP indicated with asterisks. (B) Time-lapse of a *dcd1* mutant cell cortex during phragmoplast expansion. Daggers (†) marks the edge of TAN1–YFP previously recruited in prophase and the triangle (▾) marks movement of the phragmoplast. Time stamps are in hours:minutes. (C) *Z*-projection and cortex views of wild-type and *dcd1 add1* mutant embryos in telophase. Yellow dotted lines outline the cell. (D) Representative *Z*-projections of subsidiary mother cell phragmoplasts from CIPC and DMSO control treated samples. Asterisks mark *de novo* TAN1–YFP; carets mark the expected division site. (E) Bar plots of *de novo* TAN1–YFP cell cortex accumulation in *dcd1* or *dcd1 add1* mutants, or DMSO and CIPC treated wild-type plants. Numbers above bars represent total cell numbers. *N*≥3 plants or kernels of each genotype or treatment. ***P<0.001 (Fisher's exact test). Scale bars: 10 µm.

Cytokinesis in *dcd1* mutant cells often completes in aberrant locations. That TAN1–YFP accumulated in a PPB-independent way was a surprise, because it contrasted with previously published results (Rasmussen et al. 2011; Walker et al., 2007) (*n*=21 misoriented phragmoplasts from 3 plants) (Fig. 3A,B,E). Time-lapse imaging revealed that *de novo* TAN1–YFP accumulation trailed behind the phragmoplast after it touches the cortex (Fig. 3B, *n*=22/22 cells from 3 plants, Fig. S1C). TAN1–YFP has been previously shown to accumulate near the phragmoplast midline (Martinez et al., 2017). This suggests that TAN1–YFP might be transported from the phragmoplast to the cell cortex independently from the PPB.

TAN1–YFP accumulated at the cell cortex in the *dcd1 add1* double mutant cells that never make PPBs (Fig. 3C; Fig. S2) (Wright et al., 2009). *dcd1 add1* mutants are seedling lethal, so embryos were imaged 21 days after pollination. Wild-type cells showed normal TAN1–YFP division site accumulation at all stages (100%, *n*=304 cells, *n*=24 kernels, Fig. S2A). In *fass* mutants and in cells treated with microtubule depolymerizing drugs, AtTAN::YFP is not observed at the cortex (Walker et al., 2007; Rasmussen et al., 2011). Similarly, in the *dcd1 add1* mutant, TAN1–YFP was not observed at the cortex in preprophase, prophase, metaphase or anaphase cells (0%, *n*=0/71 cells from 9 kernels; Fig. S2B). However, TAN1–YFP often accumulated at the cell cortex in telophase (72%, *n*=36/50 cells from 9 kernels). Higher resolution imaging revealed that TAN1–YFP accumulated only after the phragmoplast touched the cortex (100%, *n*=53/53 cells from 4 kernels), not before (*n*=11/11 cells from 4 kernels) (Fig. 3C; Fig. S2). TAN1–YFP rarely accumulated at the cortex ahead of the phragmoplast (4%, *n*=2/53, Fig. S3). These data further indicate that TAN1–YFP can be recruited to the cell cortex independently of the PPB.

When additional or misoriented phragmoplast arms were generated in wild-type cells using the herbicide chlorpropham (CIPC), TAN1–YFP was recruited to *de novo* cell plate fusion sites (Fig. 3D). CIPC generates branched phragmoplasts through its tubulin-binding activity but does not affect PPB formation (Liu et al., 1995; Eleftheriou and Bekiari, 2000; Buschmann et al., 2006). Wild-type cells expressing *TAN1–YFP* and *CFP–TUBULIN* were treated for 2 h with 0.7 µM or 1 µM CIPC or the respective DMSO controls and imaged. *De novo* TAN1–YFP was observed after additional or misoriented phragmoplast arms contacted the cortex (Fig. 3E, 67%, *n*=31/46 cells from 3 plants).

### Actin and the myosin XI protein O1 facilitate TAN1–YFP accumulation at *de novo* cell plate insertion sites

**Fig. 4.**
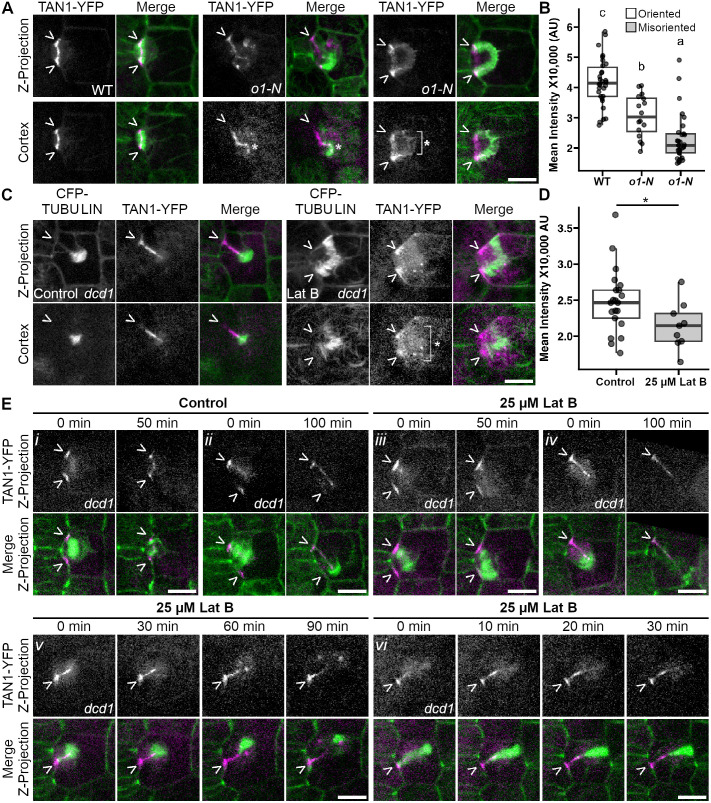
**Actin and myosin XI motor protein OPAQUE1 increase TAN1 accumulation at *de novo* cell plate insertion sites.** (A) Subsidiary cell divisions in the *opaque1* (*o1-n*) mutant and wild-type (WT) siblings. (B) Boxplot of TAN1–YFP intensities at telophase in oriented and misoriented divisions in wild type and *o1-n* mutant cells*. P*=1.02×10^–12^ (one-way ANOVA followed by Tukey's HSD, letters mark significant differences between groups). (C) TAN1–YFP accumulation in control and 25 µM LatB-treated *dcd1* mutant cells. Bracket and asterisk indicate diffuse TAN1–YFP observed in LatB treatments. (D) Boxplot of TAN1–YFP intensity at misoriented divisions of *dcd1* mutant in DMSO control (*n*=23 cells, *N*=2 plants) and 25 µM LatB (*n*=9 cells, *N*=2 plants) treatments. *P*=0.0417 (Wilcoxon rank sum test). (E) Time-lapse of *dcd1* mutant cells in control and LatB treatments. Panels display two examples each of cells at the beginning and end of control or LatB treatment: (i,ii) Sharp TAN1 accumulation in control treatment, (iii,iv) reduced TAN1 accumulation in LatB treatment, and (v,vi) lack of TAN1 maintenance with Lat B treatment. Carets (>) mark the division site and asterisks (*) mark *de novo* TAN1–YFP. Images in E are representative of *n*=15 and 17 cells in i,ii; *n*=13 and 18 cells in iii,iv; and *n*=5 and 18 cells in v,vi all from four plants. For boxplots, the box represents the 25–75th percentiles, and the median is indicated. Whiskers are for 1.5× the interquartile range from the quartile 1 and quartile 3 boundaries. AU, arbitrary units.

Accumulation of TAN1–YFP at *de novo* cell plate insertion sites is partially dependent on O1. Given that TAN1 interacts with PHRAGMOPLAST ORIENTING KINESIN1 (POK1) and POK2 (Müller et al., 2006; Rasmussen et al., 2011; Mills et al., 2022), and related kinesin 12s interact with myosin XI motor proteins (Huang et al., 2022 preprint; Nan et al., 2023), we hypothesized that O1 might be necessary for TAN1–YFP accumulation. TAN1–YFP fluorescence intensity during telophase was reduced but not absent in both correctly oriented and *de novo* cell plate fusion sites in *o1* mutants compared to wild-type siblings [Fig. 4A,B, *P*=1.02×10^−12^, one-way ANOVA followed by Tukey's honestly significant difference (HSD) test]. Therefore, O1 facilitates TAN1–YFP accumulation during telophase.

Actin filament disruption also reduced TAN1–YFP accumulation at *de novo* cell plate fusion sites. Actin filament formation was inhibited with latrunculin B (Lat B) treatment in *dcd1* mutant cells, where 10-min treatments with 25 µM Lat B inhibited actin polymerization (Fig. S4). Lat B treatment reduced TAN1–YFP accumulation at *de novo* cell plate fusion sites (Fig. 4C–E, *P*=0.0417, Wilcoxon rank sum test). To determine whether *de novo* TAN1–YFP recruitment or maintenance depends on actin filaments, 10-min time points were taken after treating *dcd1* mutant cells with control or 25 µM Lat B (Fig. 4E). We defined recruitment as accumulation of TAN1–YFP at *de novo* division sites, and maintenance as the persistence of TAN1–YFP accumulation once the phragmoplast disassembled in that location. In control-treated *dcd1* mutant cells, TAN1–YFP accumulated and remained at the cell cortex as a narrow line following the phragmoplast trajectory (*n*=15/17 cells from 4 plants, Fig. 4Ei,ii). Rarely, TAN1–YFP accumulation was reduced (*n*=1/17) or not maintained at the cell cortex (*n*=1/17). After Lat B treatments, TAN1 accumulation was often reduced (*n*=13/18, e.g. Fig. 4Eiii,iv) or not maintained after treatment (*n*=5/18, e.g. Fig. 4Ev,vi). Therefore, both TAN1–YFP recruitment and maintenance at *de novo* sites are reduced when actin filaments were disrupted.

In the absence of PPB-mediated recruitment, we observe TAN1–YFP accumulation at aberrant cell plate fusion sites that is partially dependent on actin filaments and O1. Consistent with this, when actin is disrupted in *Arabidopsis* root cells, TAN1, POK1 and myosin XI localization at the division site become diffuse (Huang et al., 2022 preprint). Actin connects the leading edge of the phragmoplast with the division site through the action of myosin VIII in *Physcomitrium patens* (Wu and Bezanilla, 2014) and is required for division plane positioning (Mineyuki and Palevitz, 1990; Gallagher and Smith, 1999; Frank et al., 2003; Gilliland et al., 2003; Galatis and Apostolakos, 2004; Facette and Smith, 2012; Vaškebová et al., 2018). During the late stages of phragmoplast expansion, actin facilitates completion of cell plate fusion (van Oostende-Triplet et al., 2017), a process potentially dependent on recruitment of TAN1 and other division site proteins. Recruitment of other division site proteins (e.g. POK1) to *de novo* cell plate fusion sites have also been observed in mutants that generate additional ectopic cell plates, suggesting that *de novo* localization might be a common feature during cytokinesis (Lebecq et al., 2023).

We hypothesize that TAN1–YFP accumulation might reflect the assembly of entire ‘division-site modules’, which could accelerate completion of cytokinesis. In the *tan1* mutant, phragmoplast disassembly at the cell cortex is significantly delayed, taking twice as long as it does in wild-type phragmoplasts (Martinez et al., 2017). Additionally, aberrantly targeted cell plates generated by CIPC treatment retain the cell-plate-specific callose polymer long after properly oriented cell plates replace callose with cellulose, indicating delays in completing cytokinesis (Buschmann et al., 2006). We hypothesize that division site proteins facilitate the rapid completion of cytokinesis, and determining how this is accomplished is a fascinating question for future research.

## MATERIALS AND METHODS

### Experimental model details

Maize (*Zea mays*) plants were grown in standard greenhouse conditions (31–33°C temperature setpoints with supplementary lighting from 17:00–21:00 at ∼400 µ E m^−2^ s^−1^) in 1 l pots with soil (20% peat, 50% bark, 10% perlite, and 20% medium vermiculite) supplemented with additional magnesium nitrate (50 ppm N and 45 ppm Mg) and calcium nitrate (75 ppm N and 90 ppm CA) and Osmocote Classic 3-4 M (NPK 14-14-14%, AICL SKU#E90550). Alternatively, plants were grown in the field (Agricultural Operations, Riverside, CA, USA; https://agops.ucr.edu/) to generate maize embryos, which were hand harvested from ears at 21–23 days after pollination.

### Method details

A full resources table is available as Table S1.

#### Plant material, genotyping and phenotyping

Plants expressing CFP–β-TUBULIN and/or TAN1–YFP (Mohanty et al., 2009; Wu et al., 2013) were genotyped with CFP–TUBULIN forward primer GFP5FOR (5′-GCGACGTAAACGGCCACAAGTTCAG-3′) and the reverse primer TubB3433R (5′-CGGAAGCAGATGTCGTAGAGC-3′) and the TAN1–YFP forward primer TAN LSP1 (5′-ACGACCGTTAGCACAGAACC-3′) and the reverse primer GFP5Rev (5′-CTGAACTTGTGGCCGTTTACGTCGC-3′) or identified by painting leaves with 4 g/l glufosinate (Finale, Bayer) in 0.1% Tween 20 (Sigma). Resistance to glufosinate was assessed after 2–5 days.

The *dcd1 add1* and *dcd1* mutants were a kind gift from Dr Amanda Wright (University of North Texas, USA). The *dcd1-mu1* and *add1* alleles were genotyped according to Wright et al. (2009) using the forward MuE2 (5′-TCCATAATGGCAATTATCTC-3′) and the reverse 55862nrev (5′-GGTGCTACATATACGCTAAAG-3′) for *dcd1-mu1* and the forward 3dCAPbfor (5′-GTTGTTTTCCCCCTTGGATT-3′) and the reverse 3dCAPbrev (5′-CTTGAGTTCTTGTTTGCTCAG-3′) for *add1*. To distinguish between wild-type and *add1* mutant alleles, PCR products were digested with the restriction enzyme KpnI overnight and then run on a 4% agarose gel for 90 min at 110 V. *dcd1* mutant plants were also identified by phenotype using glue impressions of epidermal leaf cells (Allsman et al., 2019). The *opaque1* (also known as *dcd2*) mutants were a kind gift from Dr Michelle Facette (University of Massachusetts, Amherst, USA). *o1-N1242A* mutants were identified by phenotype using a lightbox and/or glue impressions.

Leaves were dissected for imaging after 3–5 weeks of growth from the whorl until the ligule was 2 mm from the base and the abaxial epidermal cells were placed into a Rose chamber as described previously (Rasmussen, 2016) to observe dividing cells. For live imaging of wild-type and *dcd1 add1* double mutant embryos, maize plants were grown in the greenhouse or in the field under standard conditions. Ears were harvested 21–23 days after pollination. Embryos were dissected from kernels and loaded onto a Rose chamber with the flat plumule face down (Kiesselbach, 1949; see https://digitalcommons.unl.edu/ardhistrb/284/).

#### Chemical treatments

1 M CIPC (CAS 101-21-3 from TCI, #C2555) was dissolved in DMSO. Leaf samples were loaded in 0.7 µM or 1 µM CIPC or the respective 0.07% or 0.1% DMSO control in a Rose chamber and imaged after 1 to 2 h of treatment. Samples were loaded with 25 µM Lat B (Fisher Scientific, #2182-1) or the respective DMSO control. *Z*-stacks were acquired 2 h after treatment. For time-lapse imaging, samples were loaded directly into 40 µl of 25 µM Lat B and a time-lapse was started with 10-min time points. To identify what concentration of Lat B was required to depolymerize actin filaments, leaf tissue samples were treated with 0.0025 µM, 0.25 µM or 25 µM Lat B for 1 h, fixed, and stained with Alexa Fluor 488–phalloidin (Thermo Fisher Scientific, #A12379) as described previously (Nan et al., 2019).

#### Confocal microscopy

Micrographs and time-lapse data were acquired using a Yokogawa W1 spinning disk microscope with an EM-CCD camera (Hamamatsu 9100c) on a Nikon Eclipse TE inverted stand. Solid-state Obis lasers with power ranging from 40 to 100 mW were used in combination with standard emission filters (Chroma Technology). For TAN1–YFP, a 514 nm laser with emission filter 540/30 nm was used. For CFP–TUBULIN, a 445 nm laser with emission filter 480/40 nm was used. Oil or water immersion objectives (60×/1.2 NA, 100×/1.45 NA) were used. Images and time-lapses were taken with Micromanager-1.4 using a 3-axis DC servo motor controller and ASI Piezo Z stage. For time-lapse, 10 or 12 min time intervals were used as specified with *Z*-intervals ranging from 3 to 5 µm. For *Z*-stacks acquired with no time-lapse, 0.5 µm steps were used.

Images were also acquired using a Zeiss LSM 880 confocal laser scanning microscope (100× oil objective immersion lens, NA=1.46) with Airyscan super resolution mode and *Z*-intervals of 0.25 µm or 3 µm. The 0.25 µm Z-intervals were used to generate the *x-z* projection in Fig. S1C. A 514 nm-excitation laser with bandpass filters of 465–505 nm with a long-pass 525 filter was used. Images were processed using default Airyscan settings with Zen software (Zeiss).

#### Figure preparation

Figures were made using Gnu Image Manipulation Program (Gimp, version 2.10.32, https://www.gimp.org/). Image levels were only adjusted linearly and images were enlarged or rotated with no interpolation.

#### Accessions

CFP–TUBULIN and TAN1–YFP lines were generated by the Maize Cell Genomics Group (Mohanty et al., 2009). Gene sequences can be found at MaizeGDB (https://www.maizegdb.org/gbrowse) using the following accession numbers (B73, v4): *DISCORDIA 1* (Zm00001d024857), *ALTERNATIVE DISCORDIA 1* (Zm00001d010862), and *TANGLED 1* (Zm00001d038060).

### Quantification and statistical analysis

Time-lapse images, *X-Z* projections and *Z*-projections were generated using Fiji (ImageJ, http://rsb.info.nih.gov/ij/, RRID:SCR_003070). Mean fluorescence intensity was measured using the ‘straight’ or ‘oval’ tool. *X-Y* drift in time-lapses was corrected using the translation function in the StackReg plug-in in ImageJ (Thévenaz, 1998) or the Fast4DReg plugin (Laine et al., 2019). Analysis of TAN1–YFP localization and/or intensity measurements was undertaken by separating the CFP–TUBULIN channel from the TAN1–YFP channel and using the CFP–TUBULIN channel to identify the stage of cell division and location at the midplane or the cell cortex.

For Fig. 1, TAN1–YFP localization to the division site was described as ‘Normal’, ‘Faint’, ‘Uneven’ or ‘Single’ based on the presence or absence of localization and TAN1–YFP intensity at the cell midplane. Normal intensity describes wild type TAN1–YFP localization – two bright accumulations in the subsidiary mother cell that flank the guard mother cell. Faint describes two accumulations that are less intense than normal. Uneven describes two accumulations, one that is more intense than the other. Finally, Single describes cells with TAN1–YFP accumulation at one division site and absence from the other. Because there was no statistical difference between the proportion of faint classes between WT and *dcd1*, Faint was merged with the Normal class.

In Fig. 2E,F, the fluorescence intensity of TAN1–YFP was measured using a line region of interest (ROI) at the cell midplane, bisecting the region of TAN1–YFP accumulation at the division site. The number of division sites is always twice the number of cells, as, at the midplane, the division sites of the subsidiary mother cell flank the guard mother cell. For cells in prophase, the same ROI was used to measure CFP–TUBULIN accumulation in the preprophase band at the division site (Fig. 2E). When TAN1–YFP or CFP–TUBULIN accumulation was below detection as frequently observed in *dcd1* mutant subsidiary mother cell divisions, the ROI was selected at the expected division site location for a subsidiary mother cell division.

When analyzing *de novo* TAN1–YFP localization in *dcd1* or *dcd1 add1* mutants or the CIPC-treated cells in Fig. 3E, phragmoplasts were categorized as normal or aberrant, where aberrant includes misoriented phragmoplasts and split phragmoplasts in the CIPC treatments (Fig. 3E). TAN1–YFP localization was determined to be ‘normal’ if TAN1–YFP was only observed to localize to the division site, and ‘de novo’ if TAN1-YFP was observed to accumulate at *de novo* cell plate fusion sites, which were identified by observing the phragmoplast and the cell cortex.

For cortical TAN1–YFP intensity measurements in Fig. 4B and D, mean intensity was measured using a 2 µm line ROI. For misoriented phragmoplasts, ROIs were drawn starting from the leading edge of the phragmoplast along the phragmoplast midline.

Graphs, tables, and statistics were generated using the R software environment for statistical computing and graphics (https://www.R-project.org/) and Rstudio software (https://posit.co/) using the following packages: tidyr, ggplot2, ggprism and ggpubr (see https://cran.r-project.org/web/packages/index.html). Statistical details of experiments can be found in the main text and/or figure legends. Significance was defined as *P*<0.05 and parametric tests were used unless data distribution was non-normal, whereupon an equivalent non-parametric test was used instead. In Fig. 4B, the one-way ANOVA was followed by a Tukey's HSD multiple comparison test. For the comparison of categorical variables in Figs 1C and 3E, a Fisher's exact test was used.

## Supplementary Material



10.1242/joces.262097_sup1 Supplementary information
