# Supplementary material for: De novo TANGLED1 recruitment from the phragmoplast to aberrant cell plate fusion sites in maize
Source: J Cell Sci. 2024 Jun 19;137(12):jcs262097. doi: 10.1242/jcs.262097 (PMC11234383; doi:10.1242/jcs.262097)
Supplement: Supplementary information [file joces-137-262097-s1.pdf]

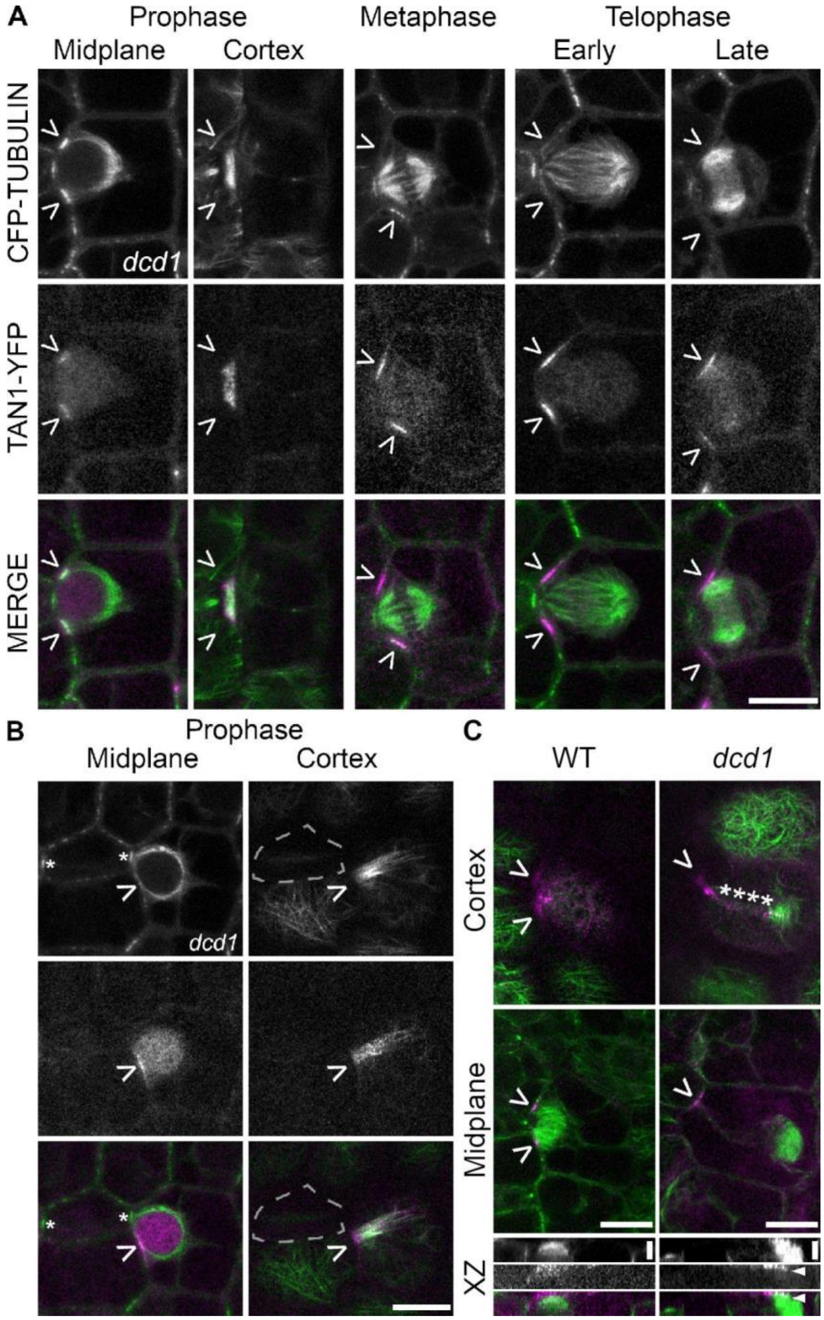

Journal of Cell Science • Supplementary information

Fig. S1. Confocal micrographs of divisions in wild type (WT) and *dcd1* plants.

(A) Representative images of correctly oriented *dcd1* divisions expressing CFP-TUBULIN (green) and TAN1-YFP (magenta) with typical microtubule structures and TAN1-YFP localization. (B) An additional example of a defective preprophase band in *dcd1* with CFP-TUBULIN and TAN1-YFP accumulation on one division site and missing from the other. Asterisks mark the typical interphase microtubule accumulation in the neighboring guard mother cell. Dotted lines outline the guard mother cell. Carets point to the division site. (C) Micrographs of wild type (left) and *dcd1* (right) cells in telophase at the cell cortex and midplane expressing CFP-TUBULIN (microtubules, green) and TAN1-YFP (magenta). Below, the CFP-TUBULIN, TAN1-YFP, and merged channels of XZ-projections showing the side view of the cell. Z-slices were taken at 0.25 µm intervals. Scale bars for A-C cortex and midplane view are 10 µm, and 3.4 µm for the XZ projections in C.

J. Cell Sci.: doi:10.1242/jcs.262097: Supplementary information

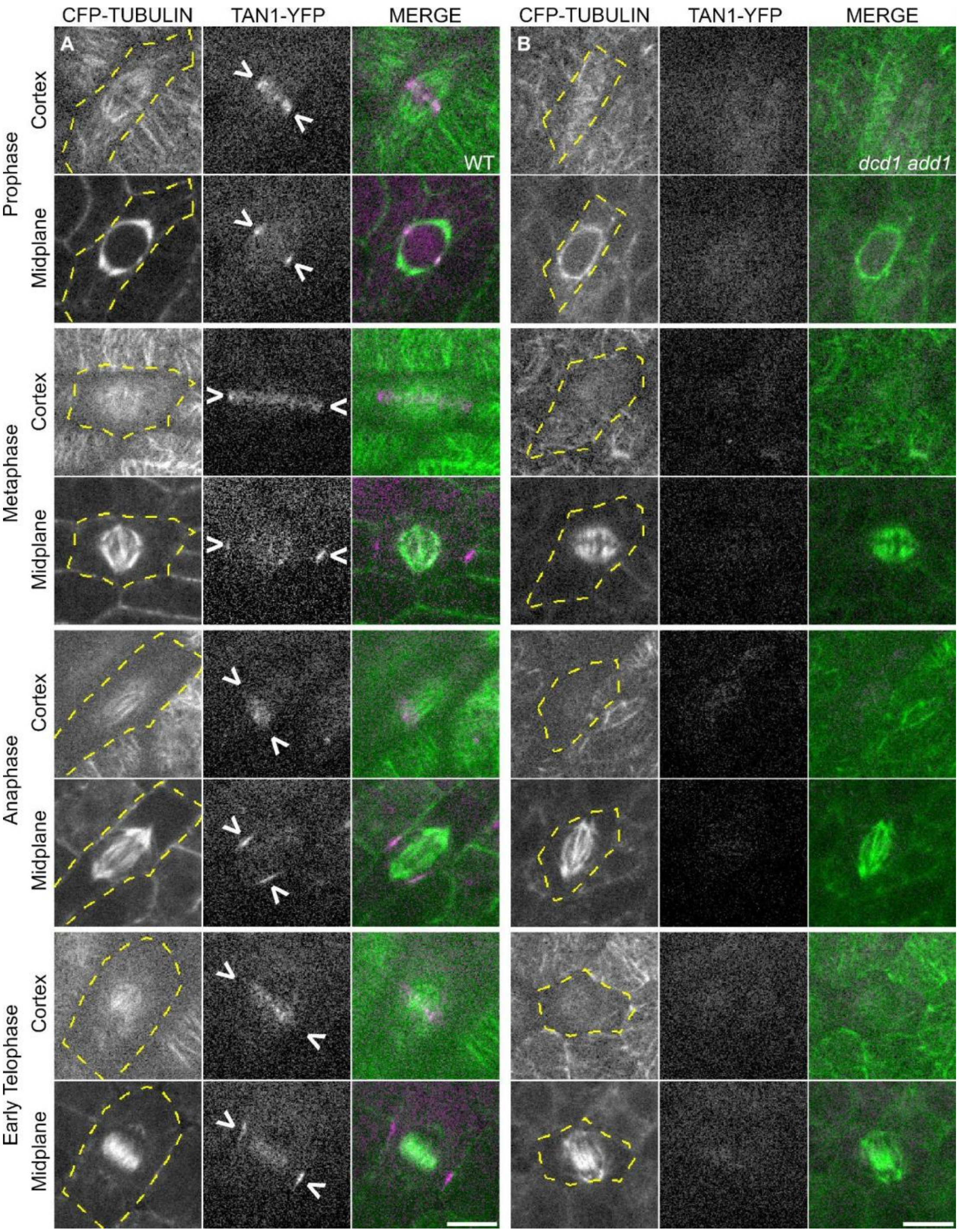

Fig. S2. TAN1-YFP localization in wild type and the *dcd1 add1* double mutant from prophase to early telophase. Micrographs of cortex and midplane views of (A) wild-type embryos and (B) *dcd1 add1* embryos expressing CFP-TUBULIN (microtubules, green) and TAN1-YFP (magenta). Arrowheads point to TAN1-YFP localization to the division site and a yellow dotted line marks the cell outline. Scale bars are 10 µm, all images in the two panels are the same magnification.

Journal of Cell Science • Supplementary information

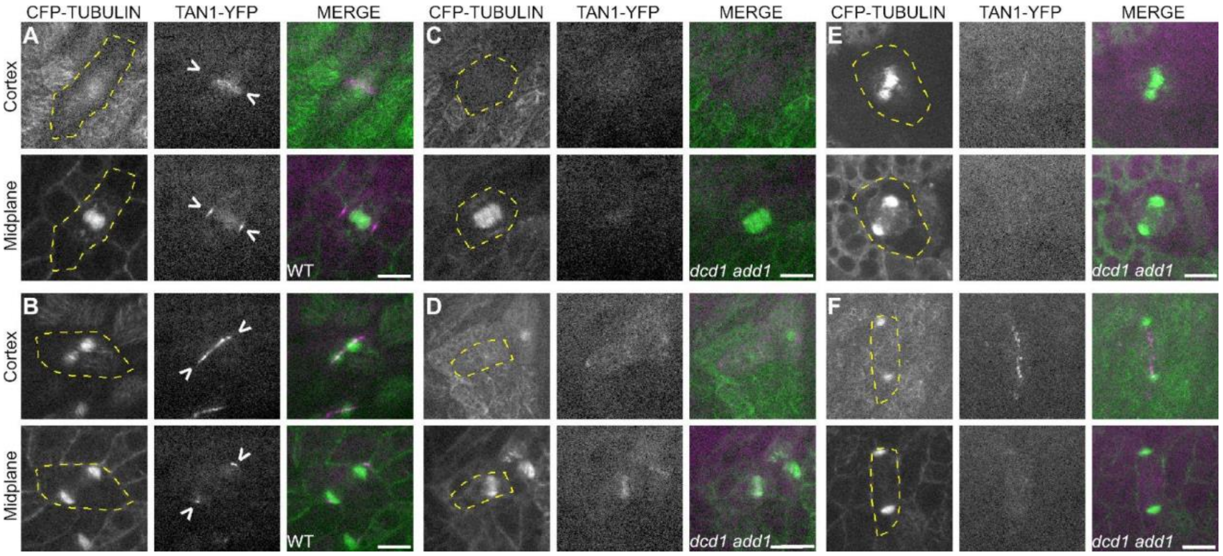

Fig. S3. TAN1-YFP localization patterns in *dcd1 add1* embryos.

(A-B) TAN1-YFP localizes to the division site (>) in wild-type embryos (A) before and (B) after the phragmoplast has reached the cell cortex. (C-D) In early telophase cells in *dcd1 add1*, TAN1-YFP is (C) absent from or (D) diffuse at the cell cortex before the phragmoplast has fully expanded. TAN1-YFP is also visible in the phragmoplast midline. (E-F) In late telophase cells in *dcd1 add1*, (E) TAN1-YFP localizes to the cell cortex as a narrow band once the phragmoplast reaches the cortex and (F) rarely localizes ahead of phragmoplast expansion. Scale bar is 10 µm.

Journal of Cell Science • Supplementary information

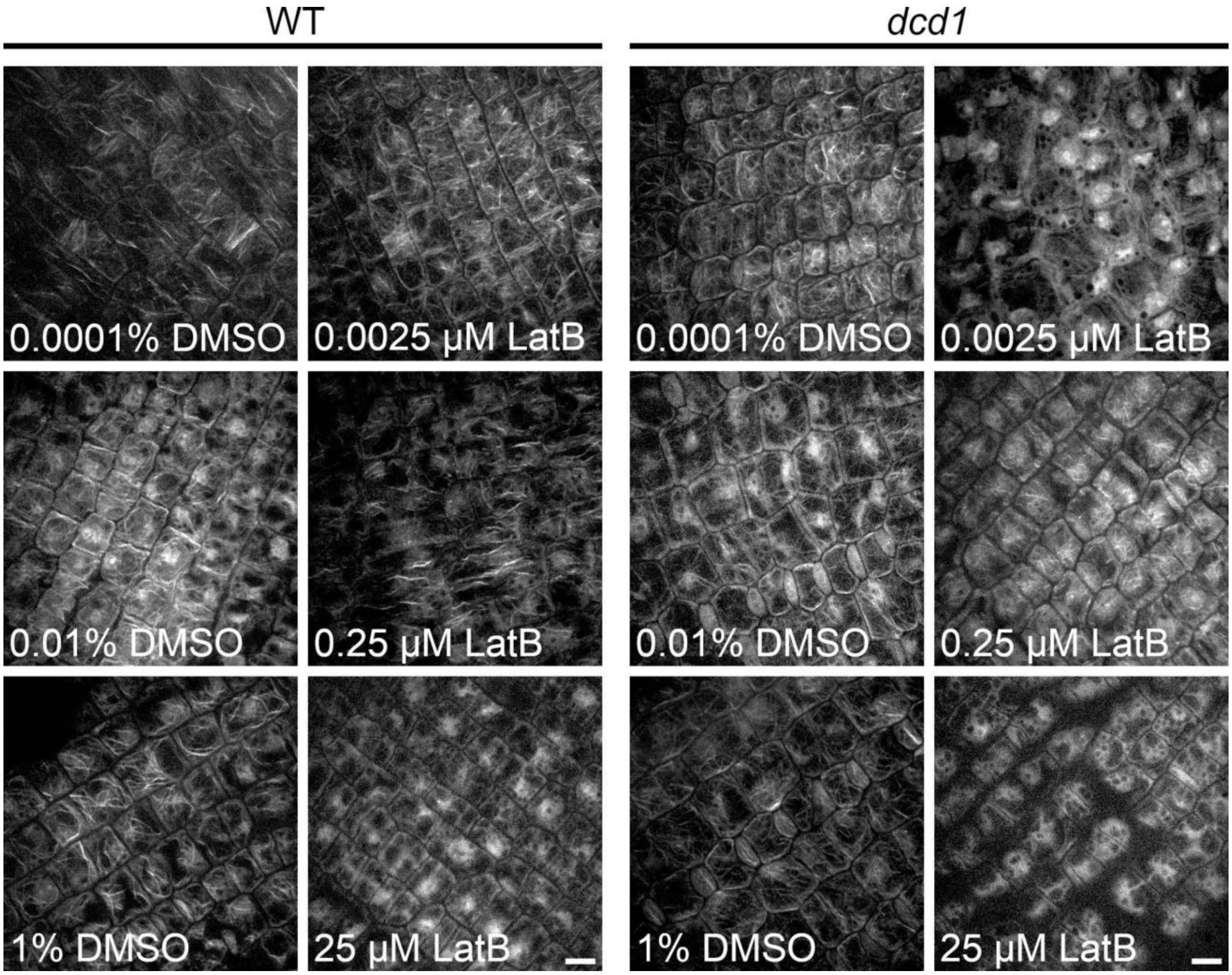

Fig. S4. Optimization of latrunculin B treatment for *dcd1* and its wild-type sibling.
Micrographs of actin filaments immunostained with Alex fluor 488-phalloidin. Scale bar is 10 µm.

Journal of Cell Science • Supplementary information

Table S1. Resources table

REAGENT or RESOURCE	SOURCE	IDENTIFIER
Chemicals, peptides, and recombinant proteins		
Chlorpropham (CIPC);	TCI	Cat#C2555; CAS:101-21-3
Latrunculin B (Lat B)	Fisher Scientific	Cat#2182-1; CAS: 76343-94-7
Dimethyl sulfoxide (DMSO)	Fisher Scientific	Cat#D128-4; CAS: 67-68-5
Alexa Fluor 488 Phalloidin	Fisher Scientific	Cat#A12379
KpnI	New England Biolabs	Cat#R3142S
Glufosinate (Finale)	Bayer	Cat#4193473
Tween 20	Fisher Scientific	Cat#BP337-500; CAS: 9005-64-5
Experimental models: Organisms/strains		
CFP-TUBULIN maize fluorescent protein tagged line	Maize Cell Genomics Group(Mohanty *et al.*, 2009); Maize Genetics Cooperation Stock Center (http://maizecoop.cropsci.uiuc.edu)	Stock #UWYO-FP019
TAN1-YFP maize fluorescent protein tagged line	Maize Cell Genomics Group(Mohanty *et al.*, 2009); Maize Genetics Cooperation Stock Center (http://maizecoop.cropsci.uiuc.edu)	Zm00001d038060 (Zm-B73 REFERENCE-GRAMENE-4.0) or Zm00001eb286860 (Zm-B73-REFERENCE-NAM-5.0); Stock #UWYO-FP017
discordia 1	Gallagher and Smith; Wright et al. 2009 (Gallagher and Smith, 1999; Wright, Gallagher and Smith, 2009)	Zm00001d024857 (Zm-B73 REFERENCE-GRAMENE-4.0) or Zm00001eb418170 (Zm-B73-REFERENCE-NAM-5.0)
alternative discordia 1	Wright et al. 2009 (Wright, Gallagher and Smith, 2009)	Zm00001d010862 (Zm-B73 REFERENCE-GRAMENE-4.0) or Zm00001eb354190 (Zm-B73-REFERENCE-NAM-

Journal of Cell Science • Supplementary information

		5.0)
opaque 1	Nan et al. 2023 (Nan, Liang, *et al.*, 2023)	Zm00001d052110 (Zm-B73 REFERENCE-GRAMENE-4.0) or Zm00001eb193160 (Zm-B73-REFERENCE-NAM-5.0)
Oligonucleotides		
Primer: CFP-TUBULIN Forward Primer (GFP5FOR) 5’-GCGACGTAAACGGCCACAAGTTCAG-3’	Integrated DNA Technologies	N/A
Primer: CFP-TUBULIN Reverse Primer (TubB3433R) 5’-CGGAAGCAGATGTCGTAGAGC-3’	Integrated DNA Technologies	N/A
Primer: TAN1-YFP Forward Primer (TAN LSP1) 5’-ACGACCGTTAGCACAGAACC-3’	Integrated DNA Technologies	N/A
Primer: TAN1-YFP Reverse Primer (GFP5Rev) 5’-CTGAACTTGTGGCCGTTTACGTCGC-3’	Integrated DNA Technologies	N/A
Primer: *dcd1-mu1* Forward Primer (muE2) 5’-TCCATAATGGCAATTATCTC-3’	Integrated DNA Technologies	N/A
Primer: *dcd1-mu1* Reverse Primer (55862nrev) 5’-GGTGCTACATATACGCTAAAG-3’	Integrated DNA Technologies	N/A
Primer: *add1* Foward Primer (3dCAPbfor) 5’-GTTGTTTTCCCCCTTGGATT-3’	Integrated DNA Technologies	N/A
Primer: *add1* Reverse Primer (3dCAPbrev) 5’-CTTGAGTTCTTGTTTGCTCAG-3	Integrated DNA Technologies	N/A
Software and algorithms		
Fiji	Schindelin et al. 2012 (Schindelin *et al.*, 2012)	https://fiji.sc/
GNU Image Manipulation Program (GIMP)		https://www.gimp.org/
R	R Core Team 2023 (R Core Team, 2023)	https://www.r-project.org/
RStudio	Posit Team 2023 (Posit team, 2023)	https://posit.co/products/open-source/rstudio/

Journal of Cell Science • Supplementary information
