# Supplementary material for: De novo TANGLED1 recruitment from the phragmoplast to aberrant cell plate fusion sites in maize
Source: J Cell Sci. 2024 Jun 19;137(12):jcs262097. doi: 10.1242/jcs.262097 (PMC11234383; doi:10.1242/jcs.262097)
Supplement: Supplementary file 1 [file joces-137-262097_review_history.pdf]

Journal of Cell Science

De novo TANGLED1 recruitment from the phragmoplast to aberrant cell plate fusion sites in maize

Aimee N. Uyehara, Beatrice N. Diep, Lindy A. Allsman, Sarah G. Gayer, Stephanie E. Martinez, Janice J. Kim, Shreya Agarwal and Carolyn G. Rasmussen

DOI: 10.1242/jcs.262097

Editor: Renata Basto

Review timeline
Original submission: 7 March 2024
Editorial decision: 22 April 2024
First revision received: 8 May 2024
Accepted: 16 May 2024

Original submission

First decision letter

MS ID#: JOCES/2024/262097

MS TITLE: De Novo TANGLED1 Recruitment to Aberrant Cell Plate Fusion Sites in Maize

AUTHORS: Aimee N Uyehara, Beatrice N Diep, Lindy A Allsman, Sarah G Gayer, Stephanie E Martinez, Janice J Kim, Shreya Agarwal, and Carolyn Rasmussen

We have now reached a decision on the above manuscript.

To see the reviewers' reports and a copy of this decision letter, please go to: https://submit-jcs.biologists.org and click on the 'Manuscripts with Decisions' queue in the Author Area. (Corresponding author only has access to reviews.)

As you will see, the reviewers gave favourable reports but raised some critical points that will require amendments to your manuscript. I hope that you will be able to carry these out because I would like to be able to accept your paper, depending on further comments from reviewers.

Please ensure that you clearly highlight all changes made in the revised manuscript. Please avoid using 'Tracked changes' in Word files as these are lost in PDF conversion.

I should be grateful if you would also provide a point-by-point response detailing how you have dealt with the points raised by the reviewers in the 'Response to Reviewers' box. Please attend to all of the reviewers' comments. If you do not agree with any of their criticisms or suggestions please explain clearly why this is so.

Reviewer 1

Advance summary and potential significance to field

Using dcd1 LOF mutant of maize (with defective PPB formation) and pharmacological interventions combined with best quliaty microscopy authors convincingly demonstrate new aspect of the TANGLED1 protein dynamics. TANGLED1 might be recruited to the abberant cortical division site de

© 2024. Published by The Company of Biologists under the terms of the Creative Commons Attribution License (https://creativecommons.org/licenses/by/4.0/).

novo from the expanding phragmoplast in the myosin-F-actin dependent manner. This is very important new insight into the dynamics of plant cell cytokoinesis.

Comments for the author

I find this report very interesting and well substantiated. The only criticism I have is to the title, abstract and introduction.
I would encourage authors to include TANGLED1 "flow" from the phragmoplast and actin dependence of this process into the title. In the Abstract it would be useful to mention dcd1 as an important tool for the study. In the introduction authors should highlight that text-book scheme of TANGLED1 recruitment to the CDZ by PPB is now fundamentally modified by this report.
However - these are suggestions. Title, abstract... whole report is well intelligible as it is.

Reviewer 2

Advance summary and potential significance to field

The manuscript submitted by Uyehara et al. provides new insight into the recruitment of TANGLED1, a plant-specific microtubule-associated protein, during asymmetric cell divisions in the maize leaf. By carefully observing and quantifying TANGLED1 recruitment during each stage of mitosis in both wild-type and mutant leaves, the authors reach two main conclusions. First, Uyehara et al. utilize the mutant divisions in dcd1 to show that defects in formation of the preprophase band (PPB) leads to division plane positioning problems. Second, the authors show that TAN1 can be recruited to the cell cortex during cytokinesis independent of its typical recruitment to the PPB and that this cortical recruitment depends, in part, on F-actin and the myosin OPAQUE1. Uyehara et al. have done an admirable job using careful observation in a large number of in vivo samples to support their conclusions. Their conclusion that accurate PPB formation is a prerequisite for proper division orientation basically supports the current thinking in the field, and it is nice to see careful data to support this model.
While the localization of TAN1 at the cortex during cytokinesis was known, this paper offers the first evidence that this population of TAN1 can be recruited de novo. To bolster this finding, it would improve the manuscript if if the authors could perform some additional measurements (with lines they already have) to begin to get at whether this de novo recruitment at the cortex is required for specific mitotic events. In summary, this is a well-written manuscript where the conclusions are supported by the provided data. After consideration of the specific points below and associated revisions, I believe the manuscript could be accepted for publication.

Comments for the author

1) As the key findings all relate to TANGLED1, it would be helpful if the introduction included a section summarizing what is already known about the protein (its localization, dynamics during mitosis, and demonstrated functions).
Including this information up front would help the general reader interpret the included data, which all revolves around TAN1. If available space is a constraint I believe that the introduction into the stomatal lineage could be slimmed down as the system needs to be introduced but most developmental steps in the lineage are not needed for interpreting the results included in this manuscript.

2) Related to Figure 1C: Could the authors clarify the reason they used separate “normal” and “faint” categories to measure “even” localization. Is there a biological significance to the two categories or do they reflect a qualitative assessment? If the difference is qualitative, I would suggest combining “normal” and “faint” into a single “even” category. If there is an underlying biological rationale for separating “normal” and “faint,” could the authors provide a clearer description of what cutoff was used in the methods?

3) Related to Figure 2F: I had trouble interpreting the graph in Figure 2F.
In particular, I had difficulty understanding “Number of division sites” on the y-axis and if that was essentially the same or different from % of observed cells.
Also, what is meant by “visible detection limit?”

© 2024. Published by The Company of Biologists under the terms of the Creative Commons Attribution License (https://creativecommons.org/licenses/by/4.0/).

4) Lines 127-129: It is unclear whether 79% of all observed cells were misoriented with lower microtubule and TAN1 recruitment or if 79% of cells with both lower microtubule and TAN1 recruitment were misoriented.

5) Line 156 – should be referring to Figure 3C, not 4C.

6) Related to Figure 4E: What are the differences between the different LatB examples and what points are being illustrated by each one? Referring to them specifically in the text or further elaboration would clarify this.

7) Because the most novel finding in the paper is that TAN1 can be recruit to the cortex independent of the PPB, it would be nice if the authors could either demonstrate a possible role for this “division-site module” (line 213) or, at a minimum, speculate about some possible functions in the discussion. While not a perfect experiment, the authors already have the lines to measure whether the rate of phragmoplast expansion or telophase duration is altered in correctly oriented divisions in o1-N (with reduced TAN1 intensity at the cortex).

First revision

Author response to reviewers' comments

A formatted PDF of this response is in the Supplementary Information. The plain text is below:

Dear Dr. Renata Basto and Reviewers,
Thank you for reading our manuscript, and making thoughtful and helpful comments to improve the manuscript. We have addressed all of your comments, and think the comments have led to a clearer and better articulated manuscript. Below you will find a point by point response to comments.

Reviewer 1 Advance summary and potential significance to field
Using dcd1 LOF mutant of maize (with defective PPB formation) and pharmacological interventions combined with best quliaty microscopy authors convincingly demonstrate new aspect of the TANGLED1 protein dynamics. TANGLED1 might be recruited to the abberant cortical division site de novo from the expanding phragmoplast in the myosin-F-actin dependent manner. This is very important new insight into the dynamics of plant cell cytokoinesis.

Reviewer 1 Comments for the Author:
I find this report very interesting and well substantiated. The only criticism I have is to the title, abstract and introduction. I would encourage authors to include TANGLED1 "flow" from the phragmoplast and actin dependence of this process into the title. In the Abstract it would be useful to mention dcd1 as an important tool for the study. In the introduction authors should highlight that text-book scheme of TANGLED1 recruitment to the CDZ by PPB is now fundamentally modified by this report. However - these are suggestions. Title, abstract... whole report is well intelligible as it is.

Thank you for these helpful comments.
We have updated the title to be:
De Novo TANGLED1 Recruitment from the Phragmoplast to Aberrant Cell Plate Fusion Sites in Maize

We have also updated the abstract to say:
Division plane positioning is critical for proper growth and development in many organisms. In plants, the division plane is established before mitosis, by accumulation of a cytoskeletal structure called the preprophase band (PPB). The PPB is thought to be essential for recruitment of division site localized proteins, which remain at the division site after the PPB disassembles. Here, we show that a division site localized protein, TANGLED1 (TAN1), is recruited independently of the PPB to the cell cortex by the plant cytokinetic machinery, the phragmoplast, using the

© 2024. Published by The Company of Biologists under the terms of the Creative Commons Attribution License (https://creativecommons.org/licenses/by/4.0/).

PPB-defective mutant *discordia1* and chemical treatments that disrupt the phragmoplast. TAN1 recruitment to de novo sites on the cortex is partially dependent on intact actin filaments and the myosin XI motor protein OPAQUE1 (O1). These data imply a yet unknown role for TAN1 and possibly other division site localized proteins during the last stages of cell division when the phragmoplast touches the cell cortex to complete cytokinesis.

*****

Reviewer 2 Advance Summary and Potential Significance to Field:
The manuscript submitted by Uyehara et al. provides new insight into the recruitment of TANGLED1, a plant-specific microtubule-associated protein, during asymmetric cell divisions in the maize leaf. By carefully observing and quantifying TANGLED1 recruitment during each stage of mitosis in both wild-type and mutant leaves, the authors reach two main conclusions. First, Uyehara et al. utilize the mutant divisions in dcd1 to show that defects in formation of the preprophase band (PPB) leads to division plane positioning problems. Second, the authors show that TAN1 can be recruited to the cell cortex during cytokinesis independent of its typical recruitment to the PPB and that this cortical recruitment depends, in part, on F-actin and the myosin OPAQUE1. Uyehara et al. have done an admirable job using careful observation in a large number of in vivo samples to support their conclusions. Their conclusion that accurate PPB formation is a prerequisite for proper division orientation basically supports the current thinking in the field, and it is nice to see careful data to support this model. While the localization of TAN1 at the cortex during cytokinesis was known, this paper offers the first evidence that this population of TAN1 can be recruited de novo. To bolster this finding, it would improve the manuscript if if the authors could perform some additional measurements (with lines they already have) to begin to get at whether this de novo recruitment at the cortex is required for specific mitotic events. In summary, this is a well-written manuscript where the conclusions are supported by the provided data. After consideration of the specific points below and associated revisions, I believe the manuscript could be accepted for publication.

Reviewer 2 Comments for the Author:
1) As the key findings all relate to TANGLED1, it would be helpful if the introduction included a section summarizing what is already known about the protein (its localization, dynamics during mitosis, and demonstrated functions). Including this information up front would help the general reader interpret the included data, which all revolves around TAN1. If available space is a constraint, I believe that the introduction into the stomatal lineage could be slimmed down, as the system needs to be introduced but most developmental steps in the lineage are not needed for interpreting the results included in this manuscript.

Thank you, we have revised the introduction to focus more on TAN1 dynamics and functions. Due to word count limits we took your advice and reduced the stomatal development section to its purely necessary components. We have also adjusted text in the results and discussion to meet the word count.

In typical land plant cell divisions, two cytoskeletal structures participate in division plane positioning: the PPB, which assembles during late G2, and the phragmoplast, which assembles during telophase and expands to complete cytokinesis. The PPB is a transient cortical ring of microtubules and actin that is an early indicator of the cell division plane (Pickett-Heaps and Northcote, 1966; Kakimoto and Shibaoka, 1987; Mineyuki, 1999; Smertenko *et al.*, 2017). Following chromosome and organelle redistribution in metaphase and anaphase, the moplast forms to facilitate cell plate formation which divides the two daughter cells (Gunning, 1982; Samuels, Giddings and Staehelin, 1995; Müller and Jürgens, 2016). The location where cytokinesis is completed is the cell-plate fusion site, and if the cell plate fuses at the location previously marked by the PPB, that location is called the division site (Smertenko *et al.*, 2017).
Genetic disruption of PPB formation often leads to significantly stunted growth, division plane positioning defects, and disrupted cortical microtubule organization which may impede cell expansion (Whittington *et al.*, 2001; Torres-Ruiz and Jürgens, 1994; Camilleri *et al.*, 2002; Kawamura *et al.*, 2006; Azimzadeh *et al.*, 2008; Wright, Gallagher and Smith, 2009; Drevensek *et al.*, 2012; Kirik, Ehrhardt and Kirik, 2012; Spinner *et al.*, 2013; Kumari *et al.*, 2021; Muroyama *et al.*, 2023).absence of >80% of PPBs generates macroscopically normal plants with minor division plane orientation defects that were attributed to spindle positioning defects (Ambrose and Cyr, 2008;

© 2024. Published by The Company of Biologists under the terms of the Creative Commons Attribution License (https://creativecommons.org/licenses/by/4.0/).

Schaefer *et al.*, 2017).PPB formation requires the PROTEIN PHOSPHATASE TYPE 2A (PP2A) phosphatase B'' regulatory subunit encoded by two related genes in maize called *discordia1 (dcd1)* and *alternative discordia1 (add1)* (Gallagher and Smith, 1999; Wright, Gallagher and Smith, 2009), homologs to *fass*/*ton2* in *Arabidopsis* (Torres-Ruiz and Jürgens, 1994; Camilleri *et al.*, 2002). In *A. thaliana* FASS/DCD1/ADD1 forms a complex with microtubule-binding proteins including TONNEAU1, TONNEAU1-RECRUITING-MOTIF proteins, and other PP2A subunits that disrupt cortical microtubule organization and PPB formation-(Wright, Gallagher and Smith, 2009; Spinner *et al.*, 2013). While *dcd1 add1* is seedling lethal and never forms PPBs, single *dcd1* mutants grow well and do not have PPB formation defects in symmetrically dividing cells (Wright et al. 2009). Instead, *dcd1* single mutants produce defective PPBs in several asymmetrically dividing cells, such as the grass-specific stomatal complex subsidiary cells, leading to division positioning defects. Subsidiary cells, generated from an asymmetric division, serve as an excellent model to analyze division-plane orientation due to consistently positioned divisions and well-characterized signaling pathways (Spiegelhalder and Raissig 2021; Gray et al. 2020) The PPB serves as a hub to recruit multiple proteins, including a small subset that remains at the division site after PPB disassembly. One division-site localized protein, TANGLED1 (TAN1), binds microtubules and is required for properly oriented divisions (Smith et al. 1996; Smith et al. 2001, Martinez et al. 2017, Martinez et al. 2020). TAN1 localization to the division site requires an intact PPB, where it is maintained until cytokinesis is completed (Walker et al. 2007; Rasmussen et al. 2011; Martinez et al. 2017). In maize, TAN1 also colocalizes with the phragmoplast midline (Martinez et al. 2017). The maize *tan1* mutant has mostly normally placed PPBs, but phragmoplast guidance defects lead to misoriented symmetric and asymmetric divisions (Smith et al. 1996; Martinez et al. 2017; Martinez et al. 2020). TAN1 promotes contact angle independent microtubule interactions which guide the phragmoplast to the division site (Bellinger et al. 2023; Martinez et al. 2020).

Here, we use the partially defective PPBs in *dcd1* mutants to measure the contribution of PPB formation to division plane positioning. To our surprise, and contrary to previous reports, the *dcd1* mutant revealed an unexpected de novo recruitment of TANGLED1 from the phragmoplast to misoriented cell plate fusion sites. We demonstrate that de novo TAN1 accumulation occurs in multiple mutants and chemically treated cells that have division plane positioning defects. Further, TAN1 accumulation is partially dependent on actin and a myosin XI OPAQUE1 (O1).

2) Related to Figure 1C: Could the authors clarify the reason they used separate "normal" and "faint" categories to measure "even" localization. Is there a biological significance to the two categories or do they reflect a qualitative assessment? If the difference is qualitative, I would suggest combining "normal" and "faint" into a single "even" category. If there is an underlying biological rationale for separating "normal" and "faint," could the authors provide a clearer description of what cutoff was used in the methods?

Thank you for this comment. During the initial characterization of TAN1-YFP localization, we qualitatively distinguished between normal and faint. TAN1 localization is initially faint in late G2 and accumulates more as the cell approaches prophase in both wild-type and *dcd1* mutant cells. Since there is no difference between faint and normal frequencies in wild-type and *dcd1* mutants, we have combined the "normal" and "faint" class into a singular "normal" class. We have updated the graph to this:

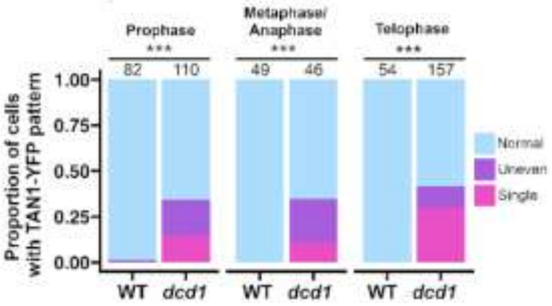

We also updated the "Quantification and statistical analysis" in the materials and methods to say:

© 2024. Published by The Company of Biologists under the terms of the Creative Commons Attribution License (https://creativecommons.org/licenses/by/4.0/).

For Figure 1, TAN1-YFP localization to the division site was described as “Normal”, “Faint”, “Uneven”, or “Single” based on the presence or absence of localization and TAN1-YFP intensity at the cell midplane. “Normal” intensity describes wild type TAN1-YFP localization- two bright accumulations in the subsidiary mother cell that flank the guard mother cell. “Faint” describes two accumulations that are less intense than “normal”. “Uneven” describes two accumulations, one that is more intense than the other. Finally, “Single” describes cells with TAN1-YFP accumulation at one division site and absence from the other.
Because there was no statistical difference between the proportion of faint classes between WT and *dcd1*, “Faint” was merged with the “Normal” class.

3) Related to Figure 2F: I had trouble interpreting the graph in Figure 2F. In particular, I had difficulty understanding “Number of division sites” on the y-axis and if that was essentially the same or different from % of observed cells. Also, what is meant by “visible detection limit?”
Time lapse imaging was done on the leaf epidermis in *dcd1*. For each dividing cell, the fluorescence intensity of TAN1-YFP at the cell midplane was measured at the division site. The number of division sites will always be twice the number of cells, as at the midplane, the division sites of the subsidiary mother cell flank the guard mother cell. We distinguished between division sites to capture the differences of TAN1-YFP fluorescence at the division site in *dcd1* cells and observe its effect on division plane orientation. The visible detection limit is the fluorescence threshold by which TAN1-YFP can be distinguished by eye above background. To make this clearer, we updated part (F) of the Figure 2 legend to:

(F) Histogram displaying the mean TAN1-YFP fluorescence intensity of cell division sites in *dcd1* colored by division orientation outcome (blue = oriented and magenta = misoriented) for time lapses that start after prophase The dotted line represents the visible detection limit or the point at which TAN1-YFP fluorescence is distinguishable over background. n = 112 cells. N = 4 plants.

We also updated the materials and methods to say:
In Figure 2E and F, the fluorescence intensity of TAN1-YFP was measured using a line ROI at the cell midplane, bisecting the region of TAN1-YFP accumulation at the division site. The number of division sites is always twice the number of cells, as at the midplane, the division sites of the subsidiary mother cell flank the guard mother cell. For cells in prophase, the same ROI was used to measure CFP-TUBULIN accumulation in the preprophase band at the division site (Figure 2E). When TAN1-YFP or CFP-TUBULIN accumulation was below detection as frequently observed in *dcd1* subsidiary mother cell divisions, the ROI was selected at the expected division site location for a subsidiary mother cell division.

4) Lines 127-129: It is unclear whether 79% of all observed cells were misoriented with lower microtubule and TAN1 recruitment or if 79% of cells with both lower microtubule and TAN1 recruitment were misoriented.
For lines 127-129, the “79%” reflects 79% of all division sites whose TAN1-YFP intensity was below the visible detection limit. The text has been updated to the following:

strongly predicts correctly oriented cell divisions. Division sites with undetectable TAN1-YFP tended to be misoriented (79%, n = 26/33 cells with TAN1-YFP fluorescence intensity at background levels, Figure 2E). For cell divisions captured

5) Line 156 - should be referring to Figure 3C, not 4C.
Thank you, we have updated the manuscript.

6) Related to Figure 4E: What are the differences between the different LatB examples and what points are being illustrated by each one? Referring to them specifically in the text or further elaboration would clarify this.

The top panels compare two examples each of control and Lat B treated *dcd1* cells at the beginning and end of treatment. The bottom two panels show more of the time lapse and show how TAN1-YFP localization changes over time with Lat B treatment. The figure legend has been updated to say:

© 2024. Published by The Company of Biologists under the terms of the Creative Commons Attribution License (https://creativecommons.org/licenses/by/4.0/).

A) Subsidiary cell divisions in the *opaque1 (o1-n)* mutant and wild-type siblings. B) Boxplot of TAN1-YFP intensities at telophase in oriented and misoriented divisions in wild type and *o1-n* mutant cells. *P* = 1.02e-12, One-way ANOVA followed by Tukey's HSD, letters mark significant differences between groups. (C) TAN1-YFP accumulation in control and 25 μM Lat B treated *dcd1* cells. Bracket and asterisk indicate diffuse TAN1-YFP observed in Lat B treatments. (E) Timelapse of *dcd1* cells in control and Lat B treatments. Panels display two examples each of cells at the beginning and end of control or Lat B treatment: (i-ii) Sharp TAN1 accumulation in control treatment, (iii-iv) reduced TAN1 accumulation in Lat B treatment, and (v-vi) lack of TAN1 maintenance with Lat B treatment. Carets (>) mark the division site and asterisks (*) mark *de novo* TAN1-YFP. Boxplot horizontal lines represent the quartiles and median. Whiskers are 1.5*IQR.

The text has also been updated to say:

To determine whether de novo TAN1-YFP recruitment or maintenance depends on actin filaments, 10-minute time points were taken after treating *dcd1* cells with control or 25 μM Lat B (Figure 4E). We defined recruitment as accumulation of TAN1-YFP at de novo division sites, and maintenance as the persistence of TAN1-YFP accumulation once the phragmoplast has disassembled in that location. In control-treated *dcd1* cells, TAN1-YFP accumulated and remained at the cell cortex as a narrow line following the phragmoplast trajectory (n = 15/17 cells, n = 4 plants, Figure 4E i-ii). Rarely, TAN1-YFP accumulation was reduced (n = 1/17) or was not maintained at the cell cortex (n = 1/17). In Lat B treatments, TAN1 accumulation was often reduced (n = 13/18, e.g. Figure 4 iii-iv) or was not maintained after treatment (n = 5/18, e.g. Figure 4E v-vi). Therefore, both TAN1-YFP recruitment and maintenance at de novo sites are reduced when actin filaments were disrupted.

7) Because the most novel finding in the paper is that TAN1 can be recruit to the cortex independent of the PPB, it would be nice if the authors could either demonstrate a possible role for this "division-site module" (line 213) or, at a minimum, speculate about some possible functions in the discussion. While not a perfect experiment, the authors already have the lines to measure whether the rate of phragmoplast expansion or telophase duration is altered in correctly oriented divisions in o1-N (with reduced TAN1 intensity at the cortex).
Thank you for this suggestion. We were wary about using the *o1* mutant because it has a strong phenotype, and we were not sure how to separate what it is doing versus what TAN1 is doing. However, we regret that we failed to articulate what we think TAN1 is doing at the last stages of cell plate fusion, which is accelerating cytokinesis. Other division site localized proteins also likely contribute. The following section has now been added to the discussion.

We hypothesize that TAN1-YFP accumulation may reflect the assembly of entire "division-site modules", which may accelerate completion of cytokinesis. In the *tan1* mutant, phragmoplast disassembly at the cell cortex is significantly delayed, taking twice as long as wild-type phragmoplasts (Martinez *et al*. 2017).
Additionally, aberrantly targeted cell plates generated by CIPC treatment retain the cell-plate-specific callose polymer long after properly oriented cell plates replace callose with cellulose, indicating delays in completing cytokinesis (Buschmann *et al*. 2006). We hypothesize that division site proteins facilitate the rapid completion of cytokinesis, and determining how this is accomplished is a fascinating question for future research.

Second decision letter

MS ID#: JOCES/2024/262097

MS TITLE: De Novo TANGLED1 Recruitment from the Phragmoplast to Aberrant Cell Plate Fusion Sites in Maize

© 2024. Published by The Company of Biologists under the terms of the Creative Commons Attribution License (https://creativecommons.org/licenses/by/4.0/).

AUTHORS: Aimee N Uyehara, Beatrice N Diep, Lindy A Allsman, Sarah G Gayer, Stephanie E Martinez, Janice J Kim, Shreya Agarwal, and Carolyn Rasmussen

I am happy to tell you that your manuscript has been accepted for publication in Journal of Cell Science, pending standard ethics checks.

© 2024. Published by The Company of Biologists under the terms of the Creative Commons Attribution License (https://creativecommons.org/licenses/by/4.0/).
